# The effect of the dual Src/Abl kinase inhibitor AZD0530 on Philadelphia positive leukaemia cell lines

**DOI:** 10.1186/1471-2407-9-53

**Published:** 2009-02-13

**Authors:** Patricia Mambou Gwanmesia, Annette Romanski, Kerstin Schwarz, Biserka Bacic, Martin Ruthardt, Oliver G Ottmann

**Affiliations:** 1 Med. Klinik II/Abt. Hämatologie, Johann Wolfgang Goethe-Universität, 60590 Frankfurt, Germany

## Abstract

**Background:**

Imatinib mesylate, a selective inhibitor of Abl tyrosine kinase, is efficacious in treating chronic myeloid leukaemia (CML) and Ph+ acute lymphoblastic leukaemia (ALL). However, most advanced-phase CML and Ph+ ALL patients relapse on Imatinib therapy. Several mechanisms of refractoriness have been reported, including the activation of the Src-family kinases (SFK). Here, we investigated the biological effect of the new specific dual Src/Abl kinase inhibitor AZD0530 on Ph+ leukaemic cells.

**Methods:**

Cell lines used included BV173 (CML in myeloid blast crisis), SEM t(4;11), Ba/F3 (IL-3 dependent murine pro B), p185^Bcr-Abl ^infected Ba/F3 cells, p185^Bcr-Abl ^mutant infected Ba/F3 cells, SupB15 (Ph+ ALL) and Imatinib resistant SupB15 (RTSupB15) (Ph+ ALL) cells. Cells were exposed to AZD0530 and Imatinib. Cell proliferation, apoptosis, survival and signalling pathways were assessed by dye exclusion, flow cytometry and Western blotting respectively.

**Results:**

AZD0530 specifically inhibited the growth of, and induced apoptosis in CML and Ph+ ALL cells in a dose dependent manner, but showed only marginal effects on Ph- ALL cells. Resistance to Imatinib due to the mutation Y253F in p185^Bcr-Abl ^was overcome by AZD0530. Combination of AZD0530 and Imatinib showed an additive inhibitory effect on the proliferation of CML BV173 cells but not on Ph+ ALL SupB15 cells. An ongoing transphosphorylation was demonstrated between SFKs and Bcr-Abl. AZD0530 significantly down-regulated the activation of survival signalling pathways in Ph+ cells, resistant or sensitive to Imatinib, with the exception of the RTSupB15.

**Conclusion:**

Our results indicate that AZD0530 targets both Src and Bcr-Abl kinase activity and reduces the leukaemic maintenance by Bcr-Abl.

## Background

The cytogenetic hallmark of chronic myeloid leukaemia (CML) and a subset of acute lymphoblastic leukaemia (ALL) is the Philadelphia (Ph) chromosome. It is a shortened chromosome 22, generated by a reciprocal translocation between chromosome 9 and 22 t(9;22)(q34;q11) [1].

The most exciting breakthrough in the treatment of Ph+ leukaemias has been the development of Imatinib as an orally bioavailable therapeutic agent [2]. Although Imatinib produces high rates of clinical and cytogenetic responses in the chronic phase of CML, the onset of resistance and clinical relapse in the advanced phases of CML and Ph+ ALL is rapid [3,4]. The main mechanisms of resistance to Imatinib include Bcr-Abl dependent mechanisms such as amplification or mutations in the Abl portion of the Bcr-Abl gene.

Recent reports have demonstrated a requirement for Src kinase activity in Bcr-Abl transformation and oncogenic signal transduction [5]. Bcr-Abl expressed in myeloid cells activates both Hck and Lyn, suggesting that these kinases might play a role in the pathogenesis of CML [6]. In Ph+ ALL, Bcr-Abl seems to stimulate different Src family kinases (SFK) such as Blk, Lck and Fyn [7].

In Imatinib resistant patients, a non-Bcr-Abl dependent up-regulation in SFK expression has been observed [8]. Up-regulation of the Src family proteins Hck and Lyn, have been shown to correlate with disease progression and resistance in cell lines and patients treated with Imatinib [9].

The NH_2_-terminal portion of Abl bears 42% identity to the SFK and shares a similar domain organisation [10]. Src inhibitors have been shown to bind to Bcr-Abl irrespective of the Abl conformation [11]. Moreover, Imatinib does not inhibit SFK directly, further supporting the possible importance of SFKs in the development of clinical Imatinib resistance [12].

Based on this rationale, we investigated the effects of a new dual Src/Abl kinase inhibitor, AZD0530 with the aim of inhibiting both Src and Bcr-Abl kinases irrespective of their conformations to explore the possibility of overcoming resistance to Imatinib with the use of AZD0530.

## Methods

### p185^Bcr-Abl ^mutant constructs

Bcr-Abl cDNAs harbouring E255K, T315I, and Y253F mutations were obtained by site-directed mutagenesis using a modification of *Stratagene's *QuickChange site-directed mutagenesis Kit protocol. For the generation of mutated plasmid DNA the following primers were used (mutated base pairs are underlined): Mut255_Fwd: 5'-G GGG CCA GTA CGGG GAA ATG TAC GAG GGC GTG-3', and Mut255_rev: 5'-CAC GCC CTC GTA CAC TTT CCC GTA CTG GC-3' (pEp185^Bcr-Abl^MutE255K); Mut315_Fwd: 5'-GTT CTA TAT CAT CAT AGA GTT CAT GAC CTA C-3' and Mut315_rev: 5'-GGT CAT GAA CTC TAT GAT GAT ATA GAA CGG-3' (pEp185^Bcr-Abl^MutT315I); and Mut253_Fwd: 5'-GGG CGG GGG CCA GTT TGG GGA GGT GTA CGA GGG C-3'and Mut253_rev: 5'-CCT CGT ACA CCT CCC CAA ACT GGC CCC CGC CCA GC-3' (pEp185^Bcr-Abl^MutY253F). Mutated plasmid DNA was sequenced using the primer Bcr-Abl 2436: 5'-CTT GAT GGA GAA CTT GTT GTA GGC-3'. All PCR-products were controlled for the presence of mutations by sequencing. The resulting cDNAs were cloned into the pENTR1A vector for further recombination into the PINCO vector as described in Beissert et al. 2008 [13] using the Gateway LR-clonase enzyme kit (*Invitrogen*, Karslruhe, Germany).

### Cell culture, Drug treatment

Cells were cultured at 37°C in 5% CO_2 _in humidified atmosphere. Human leukaemic cell lines, BV173, SEM, SupB15, and murine Ba/F3 were obtained from the German Collection of Microorganisms and Cell Cultures (DSMZ, Braunschweig, Germany). The ecotropic packaging cells Phoenix were obtained from Harald von Melchner at the Medical School of Johann Wolfgang Goethe, Frankfurt. Ba/F3 were cultured in RPMI 1640 (*Invitrogen*) supplemented with 10% fetal calf serum (FCS) (*Invitrogen*), 10ng/ml murine IL-3 (Cell Concepts, Umkirch, Germany), 1% Glutamine and 1% Penicillin/Streptomycin (40 U/ml Penicillin, 40 μg/ml Streptomycin) (*Invitrogen*). BV173, Ba/F3PINCOp185^Bcr-Abl^, Ba/F3PINCOp185^Bcr-Abl^MutE255K, Ba/F3PINCOp185^Bcr-Abl^Mut T315I, Ba/F3PINCOp185^Bcr-Abl^MutY253F were maintained in the same medium but without IL-3. SEM cells were cultured in Iscove's MDM supplemented with 10% FCS, 1% Glutamine and 1% Penicillin/Streptomycin. WTSupB15 were maintained in RPMI 1640 supplemented with 15% FCS, 1% Glutamine and 1% Penicillin/Streptomycin. RTSupB15 were cultured in supplemented RPMI 1640 medium with the addition of 1 μM Imatinib.

#### Retroviral infection

Ecotropic Phoenix packaging cells were transiently transfected with the indicated retroviral vectors as described before [14]. Transfection efficiency of the packaging cells was assessed by the detection of the percentage of green fluorescent protein (GFP) positive cells through fluorescence-activated cell sorting (FACS) analysis. Retroviral supernatant was collected at days 2 and 3 after transfection, shock-frozen in liquid nitrogen and stored at -80°C. For the infection, the retroviral supernatant was thawed on ice. Target cells were plated onto retronectin-coated (Takara-Shuzo, Shiga, Japan) non tissue culture treated 24-well plates and exposed to the retroviral supernatant for 3 hours at 37°C in the presence of 4 μg/mL polybrene (Sigma-Aldrich, Steinheim, Germany). Cells were centrifuged at 2200 rpm for 45 minutes. Infection was repeated three times and infection efficiency had to be at least 70% for each sample as assessed by the detection of GFP positive cells by FACS. Differences of transduction efficiency between the samples did not exceed 10%. For IL-3 withdrawal, cells were washed twice with PBS, and plated at 1 × 10^5^cells/ml in medium without IL-3.

### Proliferation and apoptosis assays

Viability of cells was detected by the trypan blue dye exclusion and apoptosis was determined by 7-AAD staining as described previously [15].

### Western blot

Western blot analysis was performed accordingly to widely used protocols. The following primary antibodies were used: Rabbit polyclonal antibodies included anti-phospho-c-Abl (Tyr245)-antibody (α-p-c-Abl), anti-phospho-Akt (Tyr326)-antibody (α-p-Akt), anti-Akt-antibody (α-Akt), anti-p44/p42 MAP Kinase-antibody (α-p44/42), anti-Hck-antibody (α-Hck), anti-phospho-Lyn (Tyr507)-antibody (α-p-Lyn), anti-Lyn-antibody (α-Lyn), anti-PARP-antibody (α-PARP), anti-phospho-Src family (Tyr416)-antibody (α-p-Src), Polyclonal rabbit anti-Stat5-antibody (α-Stat5). Monoclonal rabbit anti-Src (clone 36D10)-IgG-antibody (α-Src) and monoclonal mouse anti-phospho-Stat5 (Tyr694) (clone 14H2) IgG1-antibody (α-p-Stat5) (all from *Cell Signaling Technologies*, Frankfurt, Germany). Monoclonal mouse anti-c-Abl (clone 24-11)-IgG_1_-antibody (α-Abl), polyclonal rabbit anti-c-Abl (clone K-12)-IgG-antibody (α-Abl), monoclonal mouse anti-phospho-Erk (clone E-4)-IgG_2a_-antibody (α-p-Erk), polyclonal goat anti-p-Hck (Tyr 411)-IgG-antibody (α-p-Hck), polyclonal goat anti-Hck (clone N-30)-antibody (α-Hck), and polyclonal goat anti-Hck (clone M-28)-IgG-antibody (α-Hck) were purchased from *Santa Cruz *(Heidelberg, Germany). Monoclonal mouse anti-Hck (clone 18)-IgG1-antibody (α-Hck) and monoclonal mouse anti-Stat5 (clone 89)-IgG2b-antibody (α-Stat5) were from *Becton Dickinson *(Heidelberg, Germany).

Secondary polyclonal goat-anti-rabbit-IgG-HRP conjugate-antibody, polyclonal goat-anti-mouse-IgG-HRP conjugate-antibody, and polyclonal mouse-anti-goat-IgG-HRP conjugate-antibody were purchased from *Dianova GmbH*, (Hamburg, Germany).

### Statistical analysis

Data were compared by a two-tailed Student *t *test; *p *values < 0.05 were considered to be significant.

## Results

### AZD0530 specifically blocks proliferation of Ph+ cells

**Figure 1:**
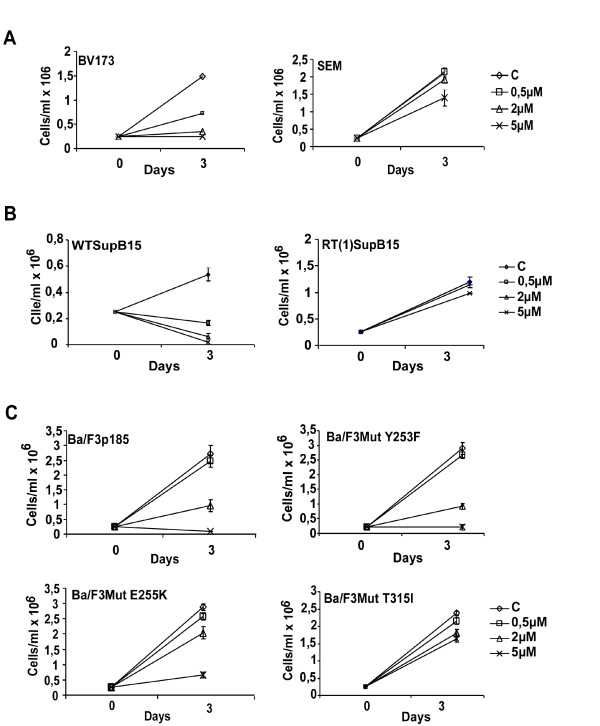
**Effects of AZD0530 on proliferation**. **A **AZD0530 specifically blocks the proliferation of Ph+ BV173 cells in a dose dependent manner, while the proliferation of the Ph- SEM cells is not markedly affected. **B **AZD0530 inhibited proliferation of WTSupB15, in a dose-dependent way. No effect was observed on the proliferation of RTSupB15 upon treatment with AZD0530. **C **AZD0530 inhibited the proliferation of Ba/F3 cells expressing WTp185^Bcr-Abl ^and the Bcr-Abl Mut Y253F. Ba/F3 cells expressing the Bcr-Abl Mut E255K were less sensitive to AZD0530 as compared to Mut Y253F and cells expressing Bcr-Abl Mut T315I were stable in the presence of AZD0530. All cell lines were treated with the indicated concentrations of AZD0530 for three days and proliferation was determined by trypan blue exclusion of viable cells. Results are the mean values of 3 independent experiments carried out in duplicates +/- S.D.

To determine the effect and specificity of AZD0530 on Src and Bcr-Abl mediated growth inhibition of Ph+ cells, the CML blast cell line BV173, was treated with various concentrations of AZD0530, and cell proliferation was measured by trypan blue exclusion of viable cells. As control for specificity the Ph- ALL cell line SEM, was used. Figure 1A shows that incubation with AZD0530, resulted in a dose-dependent decrease in cell proliferation of BV173 cells in contrast to the SEM cells over a three-day incubation period. In the BV173 cells, growth inhibition could be observed as from 0,5 μM AZD0530 when compared to DMSO treated cells. Proliferation in the SEM cells was not affected in the presence of 5 μM AZD0530 when compared to control cells.

These data show that AZD0530 is able to specifically block growth of Ph+ patient derived cells.

### AZD0530 does not overcome resistance to Imatinib in the RTSupB15

Clinical relapse and resistance to Imatinib has been shown to develop rapidly in the advanced phases of CML and Ph+ ALL patients mainly due to Bcr-Abl-dependent mechanisms such as amplification or mutations in the Abl portion of the Bcr-Abl gene. To examine the role of other potential mechanisms of Imatinib-resistance, the Imatinib-resistant Ph+ ALL cells RTSupB15 were established by long term culture of WTSupB15 cells with increasing amounts of Imatinib [16]. The RTSupB15 were viable and grew well in the presence of 1 μM Imatinib as compared to the parental WTSupB15 cells (data not shown). Treatment of RTSupB15 with Imatinib led to the down-regulation of the Bcr-Abl activity suggesting a mechanism independent of the kinase activity of Bcr-Abl. In RTSupB15 cells, no mutations were detectable in Bcr-Abl exons 4, 6, and 7 upon analysis. Cytogenetically WTSupB15 and RTSupB15 presented no differences [16]. As shown in Figure 1B, proliferation of the parental WTSupB15 cells was blocked in the presence of AZD0530, when compared to the Imatinib resistant cell line RTSupB15 that did not respond to treatment with AZD0530.

In summary, these data show that AZD0530 is unable to overcome non-mutational Imatinib resistance.

### AZD0530 blocks the proliferation of p185^Bcr-Abl ^expressing Ba/F3 cells as well as Bcr-Abl expressing Ba/F3 cells harbouring mutations which induce Imatinib resistance

Recent data have demonstrated that Bcr-Abl-dependent cell lines are sensitive to growth arrest induced by dual Src/Abl kinase as well as Src selective kinase inhibitors such as Dasatinib, PP2 and A-419259 [5]. To investigate the effect of AZD0530 on cells that depend on Bcr-Abl for their survival we treated Ba/F3 cells, which were rendered factor-independent by the expression of Bcr-Abl with AZD0530. Empty vector transduced Ba/F3 cells grown in the presence of IL-3 were not inhibited upon exposure to AZD0530 (Data not shown). In contrast Ba/F3p185^Bcr-Abl ^showed a concentration-dependent growth inhibition.

Taken together these data show that AZD0530 targets specifically the Bcr-Abl dependent signalling. To investigate the effects of AZD0530 on Bcr-Abl harbouring mutations conferring Imatinib-resistance (Y253F, E255K and T315I) Ba/F3 cells expressing these mutants were treated with the dual Src/Abl kinase inhibitor AZD0530, and proliferation was assessed comparing them with the Ba/F3 infected p185^Bcr-Abl ^cells treated in a similar manner (Figure 1C). Here we show that proliferation of p185^Bcr-Abl ^and Mut Y253F was inhibited by the use of AZD0530 (Figure 1C, see Additional files 1 and 2). Mut E255K was less sensitive to AZD0530 as compared to Mut Y253F, and needed higher concentrations of the inhibitor demonstrated by an altered dose-response with Mut E255K cells. Proliferation of Mut T315I was not affected by the presence of AZD0530.

Taken together these results indicate that AZD0530 is able to overcome resistance of Bcr-Abl Mut Y253F and E255K but not of T315I.

### AZD0530 specifically induces apoptosis in Ph+ cells

**Figure 2:**
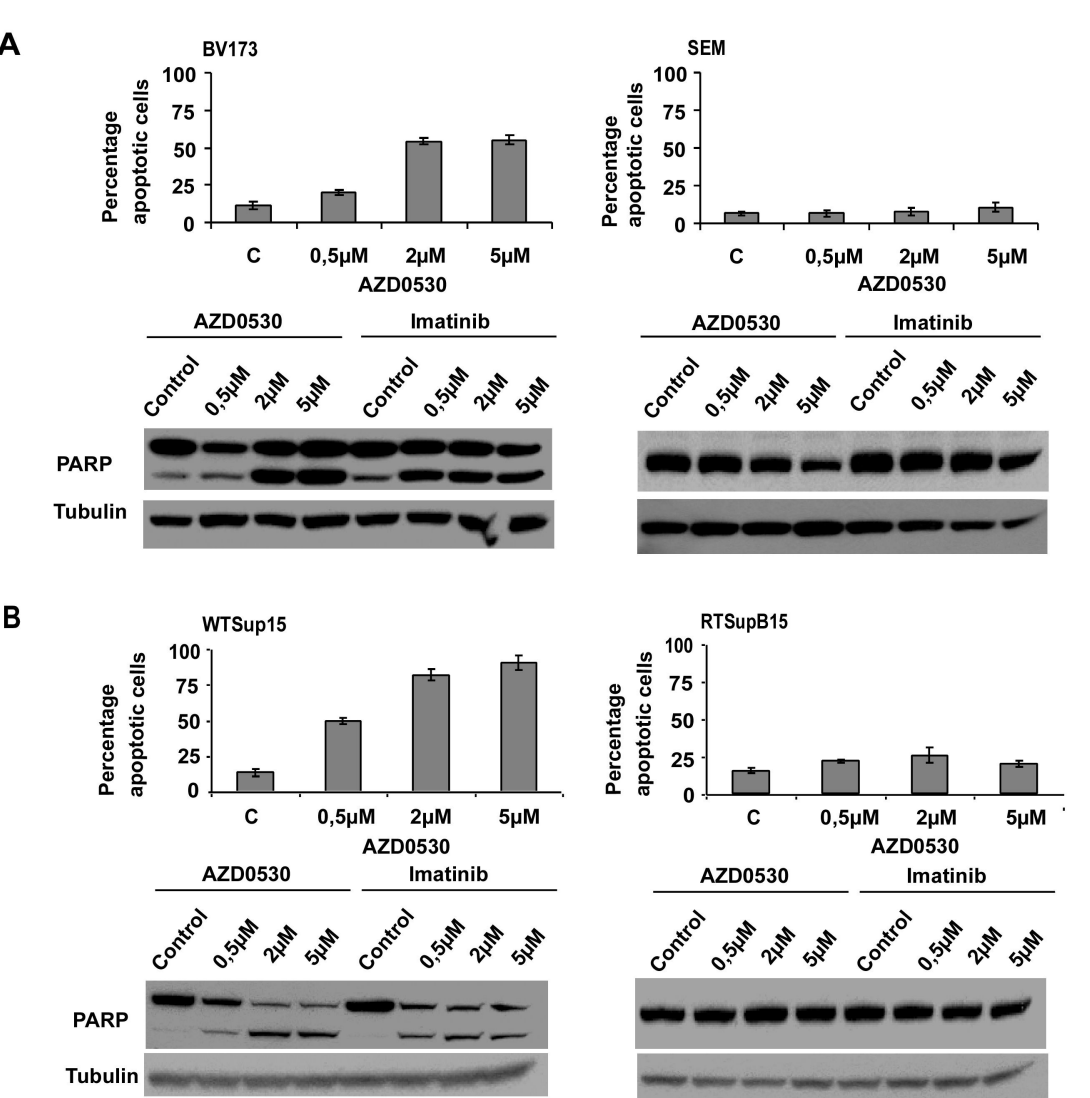
**Apoptosis induction by AZD0530**. **A **Inhibition of SFK and Bcr-Abl by AZD0530 and Imatinib leads to a Bcr-Abl-dependent induction of apoptosis, as seen in BV173 (Ph+) versus SEM (Ph-) cell lines. One representative experiment out of 3 is given. **B **Induction of apoptosis in WTSupB15 cells and not in RTSupB15 cells. All four cell lines (BV173 (Ph+), SEM (Ph-), WTSupB15 (Ph +) and RTSupB15 (Ph+ Imatinib resistant)) were treated with increasing concentrations of both compounds for three days. The percentage of apoptotic cells was measured by staining with 7-AAD. Results represent the mean values of three independent experiments +/- SD. For PARP cleavage, cells were treated for three days and immunoblotted. Intact (116 kDa) and cleaved (85 kDa) PARP are shown. Equal protein loading was monitored by probing tubulin on the same membrane. One representative experiment out of 3 is given.

To answer the question whether the dose-dependent inhibition of Ph+ cell proliferation by AZD0530 was associated with the induction of apoptosis, both BV173 (Ph+) and SEM (Ph-) cell lines were treated in parallel with increasing concentrations of AZD0530 and Imatinib for three days and apoptosis was measured by 7-AAD staining. At the protein level, poly(ADP-ribose)polymerase (PARP) cleavage was used as a sign of apoptosis, and was examined in whole cell lysates by immunoblotting. As shown in Figure 2A, BV173 cells underwent a dose-dependent induction of apoptosis of 20%, 54% and 55% in the presence of 0.5 μM, 2 μM and 5 μM AZD0530 respectively. In contrast to BV173 cells, only 11% induction of apoptosis was reached in the SEM cells, even at the highest concentration of 5 μM AZD0530. In the SEM cells, neither AZD0530 nor Imatinib induced significant PARP cleavage, whereas in BV173 cells, PARP was already cleaved in the presence of 0.5 μM AZD0530 and 0.5 μM Imatinib. The induction of PARP cleavage in the BV173 cells correlated well with apoptosis measurement.

These results confirmed that the inhibition of SFKs and Bcr-Abl by both compounds was associated with the induction of apoptosis and was Bcr-Abl dependent.

### AZD0530 does not induce apoptosis in Imatinib resistant RTSupB15 cells

To further investigate the influence of AZD0530, apoptosis measurement and PARP cleavage were assessed in the RTSupB15 cells and the results were compared to that of the parental WTSupB15 cell line (Figure 2B). In the WTSupB15 cells, apoptosis measurement by 7-AAD staining (IC_50 _of 0.5 μM) correlated well with PARP cleavage (bottom panel), with a strong effect of 2 μM ADZ0530. This is contrary to the RTSupB15 cells with not more than 20% of cells undergoing apoptosis at the highest concentration (5 μM AZD0530) used, as compared to the control cells. This could be confirmed by a lack of PARP cleavage (bottom panel).

### AZD0530 inhibits SFK activity at concentrations that cause growth arrest and induce apoptosis in Bcr-Abl positive cells

**Figure 3:**
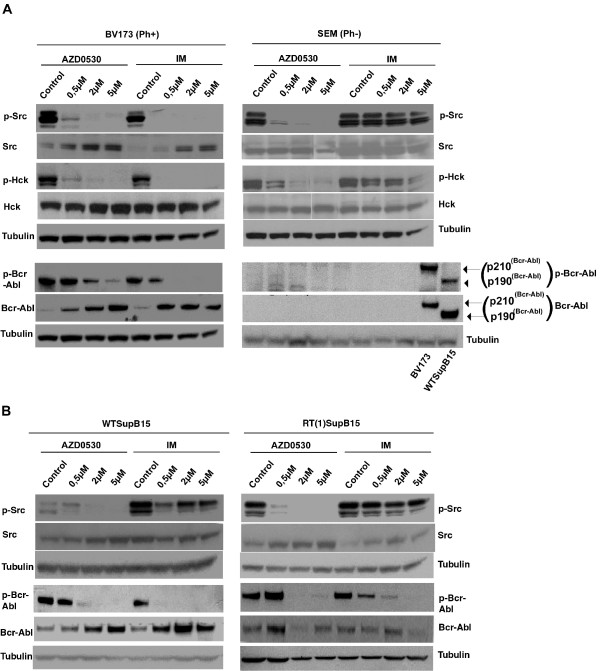
**Cell-type-specific effect of AZD0530**. **A **BV173 (Ph+) and SEM (Ph-) cells were incubated with the indicated concentrations of AZD0530 and Imatinib for three days. Whole cell lysates were probed for activated SFKs, phosphorylated Hck and the activated form of Bcr-Abl. BV173 and WTSupB15 samples in the SEM (Ph-) panel were used as a control for the detection of Bcr-Abl and phosphorylated Bcr-Abl. Tubulin was used as control for protein loading. (1) In BV173 cells SFK activity but not Bcr-Abl activation was inhibited at concentrations that cause growth arrest and induce apoptosis. (2) In BV173 cells, SFKs were shown to act downstream of Bcr-Abl. **B **Bcr-Abl as substrate of SFKs. WTSupB15 (left) and RTSupB15 (right), were treated with AZD0530 and Imatinib for three days. Whole cell lysates were prepared and probed for activated SFKs, phosphorylated Hck and the activated form of Bcr-Abl. Tubulin was used as a control for equal protein loading. In both cell lines Bcr-Abl was shown to be transphosphorylated by SFKs.

To determine the effects of AZD0530 and Imatinib on SFK activity in the CML cell line BV173 cells were exposed to the same inhibitor concentrations used in the proliferation and apoptosis assays (Figure 3A). Whole cell lysates were analysed by Western blot using an antibody specific for the activated form of the Src family of tyrosine kinases. Lysates were additionally probed for phosphorylated Bcr-Abl, to further investigate if the activation status of Bcr-Abl was correlating with growth inhibition and apoptosis. SEM cells were used as control. Figure 3A shows that in BV173 cells 0.5 μM AZD0530 and 0.5 μM Imatinib strongly inhibited both Src phosphorylation of all SFKs and Hck phosphorylation, but higher concentrations of both inhibitors were needed to inhibit phosphorylation of Bcr-Abl. Inhibition of SFK phosphorylation and Hck phosphorylation by AZD0530 in BV173 cells was seen at concentrations which led to apoptosis induction and reduced cell proliferation.

In contrast SFK and Hck inhibition in SEM cells was observed in the presence of AZD0530 but not Imatinib, and neither led to growth inhibition nor apoptosis induction.

### Bcr-Abl is up-stream to SFK in CML-BV173 cells but down-stream in ALL-SupB15 cells

In Ph+ leukaemic cells, it is not clear if the interaction between Bcr-Abl and SFKs is solely due to activation of Src kinases by Bcr-Abl, activation of Bcr-Abl by SFKs or, if there exists a transphosphorylation mechanism between both tyrosine kinases.

To investigate the influence of Abl kinase inhibition on SFK activation we exposed BV173 cells to Imatinib. Figure 3A shows that treatment of BV173 with Imatinib led to a complete dephosphorylation of both Bcr-Abl and SFKs (in particular Hck). These findings suggest that the Src kinases are a substrate of Bcr-Abl in BV173 cells. A similar phenomenon was seen in Imatinib resistant Ba/F3MutT253F cells (see Additional file 2).

It has already been shown that Hck, Lyn, Fyn and Fgr each bind the kinase domain, at the C-terminal tail, and SH3/SH2 region of Bcr-Abl [5]. It is known that the Bcr-Abl-Hck interaction is independent of catalytic activity [17]. Furthermore, Hck, Lyn and Fyn strongly phosphorylate recombinant, purified Abl SH3-SH2 protein *in vitro *[5]. These findings provide an argument for the fact that SFKs interact with and promote Bcr-Abl leukaemogenesis. This hypothesis was investigated in Imatinib sensitive and resistant cells. To this effect, the Imatinib resistant cell line RTSupB15 and its parental wild type cell line WTSupB15 were exposed to AZD0530 and Imatinib (Figure 3B). Whole cell lysates were probed with antibodies to the activated forms of Bcr-Abl and Src kinases. In both cell lines, activated Bcr-Abl was inhibited by both compounds, but higher concentrations of Imatinib were needed in the RTSupB15 cells when compared to WTSupB15 cells. The SFKs were dephosphorylated by AZD0530 but not by Imatinib in both cell lines (WTSupB15 and RTSupB15). This provides evidence that Bcr-Abl acts downstream of Src kinases in RTSupB15 as well as WTSupB15 and explains why dephosphorylation of Bcr-Abl did not affect the activity of the Src kinases.

### AZD0530 blocks the activation of Stat5, Erk and PI3K/Akt specifically in Bcr-Abl positive cells

**Figure 4:**
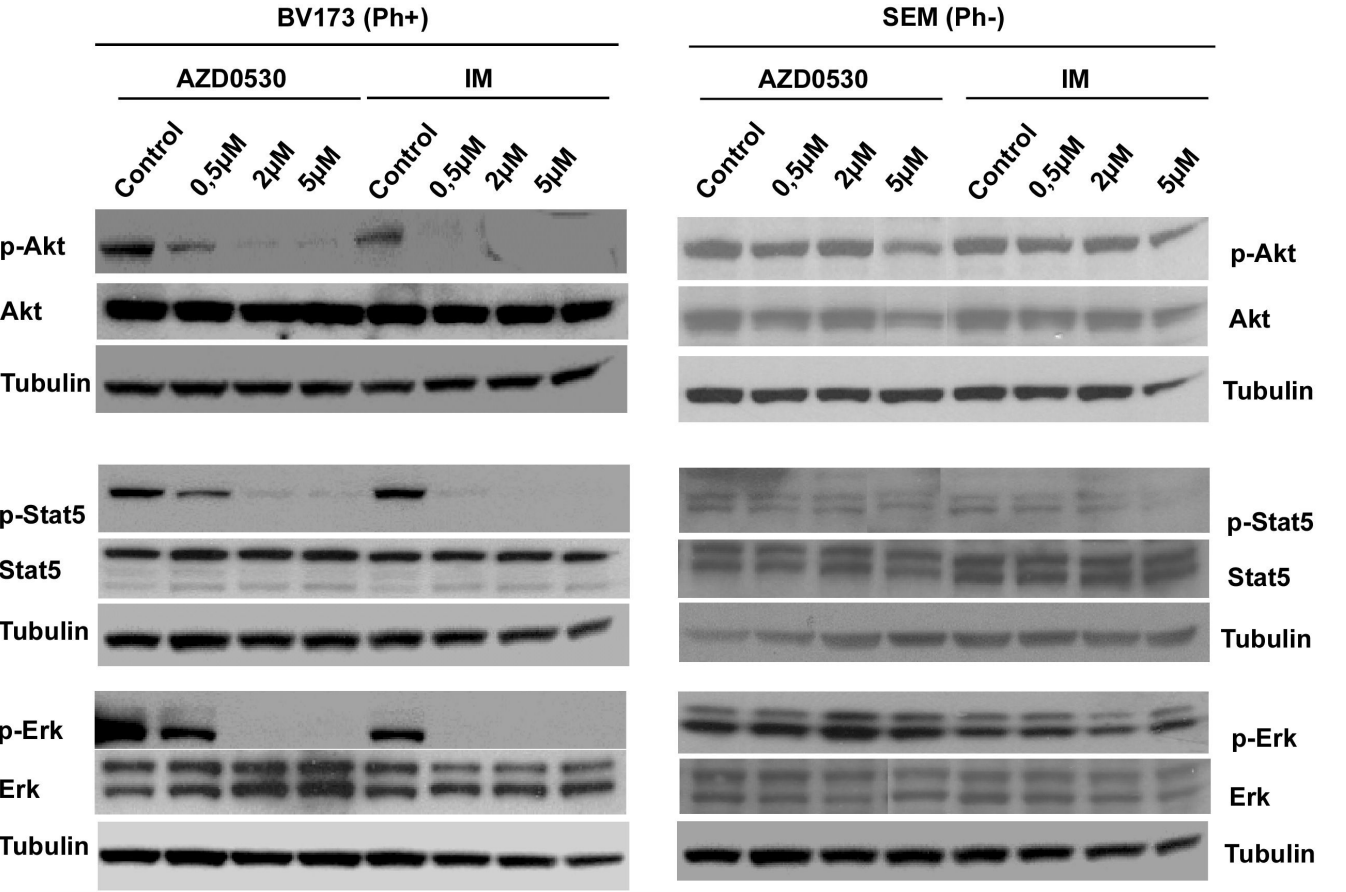
**AZD0530 inhibits survival signalling pathways involved in Bcr-Abl leukaemogenesis**. Whole cell lysates from AZD0530 and Imatinib treated BV173 cells (left) and SEM cells (right) were probed with antibodies (phosphorylated and non-phosphorylated forms) against Akt, Stat5 and Erk. Specificity of both inhibitors to Bcr-Abl was tested by comparing results of BV173 and SEM cells treated under similar conditions. In BV173 cells the activation of Akt, Stat5 and Erk was inhibited by AZD0530 and Imatinib, in contrast to SEM cells which showed no regulation of all three proteins. One representative experiment out of 3 is given.

Activation of the Raf/Mek/Erk, PI3K/Akt and Stat pathways synergize to promote Ph+ leukaemic cell growth. To investigate if inactivation of Src kinases and Bcr-Abl by AZD0530 could interfere with the leukaemic survival signalling pathways, lysates from BV173 cells treated with AZD0530 or Imatinib were probed with antibodies (activated and whole protein) against Stat5, Erk, and Akt kinases. Results were compared to Ph- SEM cells (Figure 4). In the BV173 cells, both compounds inhibited phosphorylation of Stat5, Erk and Akt kinases. This correlated well with the stronger effect on the inhibition of SFKs. These results support the idea that the Src kinases couple Bcr-Abl to its substrate proteins, and AZD0530 may target more specifically the Src kinases. Neither AZD0530 nor Imatinib affected the activation of all three proteins in the SEM cells.

### Combining AZD0530 with Imatinib has an additive effect on BV173 cells

**Figure 5:**
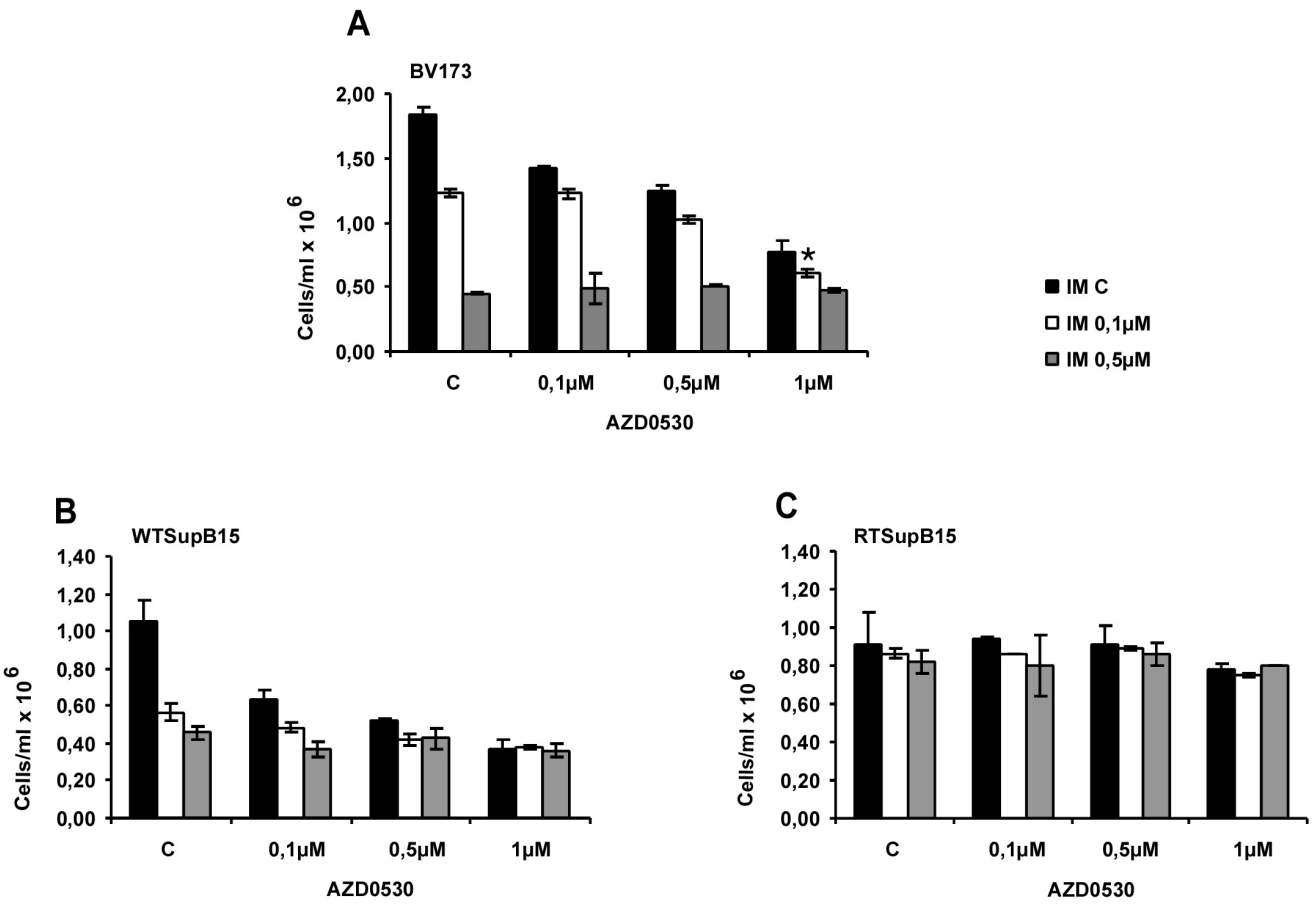
**Anti-proliferative effect of AZD0530 in combination with Imatinib**. Effects of AZD0530 and Imatinib as single agents or in combination on the proliferation of BV173 (A), parental WTSupB15 (B) and Imatinib resistant RTSupB15 (C) cell lines. Combining AZD0530 with Imatinib had an additive effect only in BV173 cells. All three cell lines were plated in duplicates at the indicated concentrations, and proliferation was assessed after three days by dye exclusion of viable cells. Results represent the mean of 3 independent experiments +/- S.D.

Combination therapy of Imatinib with an inhibitor of Imatinib-resistant Bcr-Abl is frequently used to prevent emergence of resistance. Dual Src/Abl kinase inhibitors exhibit outstanding *in vitro *inhibitory profiles against Imatinib resistance due to Bcr-Abl mutations and resistance independent of Bcr-Abl [11]. To evaluate this strategy, cells were treated with combinations of AZD0530 (0.1 μM, 0.5 μM, 1 μM) and Imatinib (0.1 μM, 0.5 μM) and compared to the untreated control cells (Figure 5). Growth inhibition was analysed using proliferation assays as described previously [15]. An additive effect was seen for BV173 cells, using 0.1 μM Imatinib and 1 μM AZD0530 (0.6 × 10^6 ^cells/ml) compared to the single agents alone (0.1 μM Imatinib (1.23 × 10^6 ^cells/ml) and 1 μM AZD0530 (0.8 × 10^6 ^cells/ml))(Figure 5A). No major difference was seen upon treatment of the ALL cell lines WTSupB15 (Figure 5B) and RTSupB15 (Figure 5C) between single agents and combinations of AZD0530 and Imatinib.

## Discussion and conclusion

Recent reports have demonstrated a requirement for Src kinase activity in Bcr-Abl transformation and oncogenic signal transduction [5].

Here, we present AZD0530, a new, orally administered, potent and highly selective dual Src/Abl kinase inhibitor, whose effects have been investigated in solid tumours [18]. In this study, we demonstrated that AZD0530 specifically inhibits the growth of CML cells and Ph+ ALL cells in a dose dependent manner, but had little or no effect on Ph- ALL cells. AZD0530 inhibited the growth of Imatinib resistant Ba/F3 expressing MutY253F and MutE255K cells. This could be explained by the fact that substitution of Threonine by Phenylalanine (MutY253F) and Glutamine by Lysine (MutE255K) distorted the conformation needed by Imatinib to attach to Bcr-Abl in this cells, but such a conformation was not needed by AZD0530. This indicates that this compound could be used to overcome resistance to Imatinib due to mutations, in Ph+ cells.

A significant induction of apoptosis in the Ph+ ALL cell line WTSupB15 occurred at lower concentrations (0.5 μM) when compared to the CML cell line, BV173 (2 μM). It could be concluded that the SFK inhibited by AZD0530, contribute to a greater extent to proliferation and survival in Ph+ ALL cells than the CML cells. Neither proliferation nor apoptosis induction was influenced by AZD0530 in the Imatinib resistant RTSupB15 cells (Ph+ ALL). This confirmed that resistance to Imatinib in these cells is not Src dependent.

Analysis of the mechanisms of action of AZD0530 showed that higher concentrations of AZD0530 and Imatinib were needed to inhibit the activation of Bcr-Abl, when compared to the concentrations needed to inhibit activated SFK in BV173 cells. Growth and survival of the Ph- ALL cell line SEM was not influenced by both compounds, though AZD0530 inhibited the activation of SFKs in these cells. This also confirmed that Src kinases influence the survival of Bcr-Abl positive cells.

In this study, it could not be clearly defined if the inhibitory effects of AZD0530 were a result of its direct effect on Src kinases, Bcr-Abl or on both kinases. Since Src kinases are inhibited by Imatinib in BV173 cells but not in Ph- SEM cells, it could be hypothesized that SFKs are transphosphorylated and acting downstream of Bcr-Abl in the CML blast cell line BV173. In contrast to the BV173 cells, in the SupB15 cells, Bcr-Abl was described as acting downstream of SFKs. In SupB15 cells, AZD0530 down-regulated the activated forms of both SFK and Bcr-Abl, in contrast to Imatinib, which inhibited only the activity of Bcr-Abl, and not SFK. This can be explained by the fact that inhibition of Bcr-Abl alone (by Imatinib) has no effect on SFK. Hence Bcr-Abl can only be activated by SFK in this cell line and not vice versa.

In BV173 cells, both AZD0530 and Imatinib blocked Erk, Akt and Stat5 activation at concentrations that inhibited SFKs but did not affect Bcr-Abl tyrosine phosphorylation. This was an indication that SFKs coupled Bcr-Abl to its survival and signalling molecules, enhancing disease progression. This was in contrast to SEM cells in which AZD0530 inhibited the activation of SFKs but not the activation of Erk, Akt and Stat5. This confirmed that the activity of these substrate proteins was Src independent in Ph- cells.

Treatment of the BV173 cells but not the WTSupB15 cells with a combination of AZD0530 and Imatinib yielded an additive antiproliferative effect, when compared to the single agents alone. Taken together these data suggest an additional effect of AZD0530 on Imatinib in BV173 cells that seems to be cell type specific.

In advanced Ph+ leukaemia (Ph+ ALL or CML blast crisis) clinical trials with Imatinib have shown that Bcr-Abl inhibition is not sufficient to prevent disease progression or to restrict expansion of resistant cells. This has necessitated the development of treatment regimens, where single compounds target both Bcr-Abl and its substrate proteins as seen in the case of AZD0530.

In recent investigations we have been able to show that in primary patient cells resistant to Imatinib, Src kinase inhibition is more effective in the presence of AZD0530 when compared to the Imatinib treated cells. In summary, our *in vitro *data strongly suggest that inhibition of SFK augments growth inhibition achieved by Bcr-Abl kinase inhibitors. Two of the most frequent domain mutations, which show resistance to Imatinib (MutY253F and E255K), responded to treatment with AZD0530.

## Competing interests

The authors declare that they have no competing interests.

## Authors' contributions

PMG – carried out most of the experiments and drafted the manuscript. AR – participated in the design of the study and contributed to write the paper. KS – performed experiments and statistical analysis. BB – performed experiments and provided experimental tools. MR – conceived of the study, participated in its design and contributed to write the paper. OGO – conceived of the study and participated in its design and coordination. All authors read and approved the final manuscript.

## Pre-publication history

The pre-publication history for this paper can be accessed here:

http://www.biomedcentral.com/1471-2407/9/53/prepub

## Supplementary Material

Additional file 1 **AZD0530 overcomes Imatinib resistance and induces growth arrest in Imatinib resistance Ba/F3MutT253F cells.** Ba/F3MutT253F cells were grown in the presence of AZD0530 (left) and Imatinib (right) for three days. Proliferation was assessed by trypan blue exclusion of viable cells, and the percentage of apoptotic cells was measured by staining with 7-AAD. Results are the mean of 3 independent experiments carried out in duplicates +/- S.D. Click here for file

Additional file 2 **AZD0530 inhibits Bcr-Abl activation and its downstream signalling pathways in Imatinib resistance Ba/F3MutT253F cells.** The Imatinib resistant cell line Ba/F3MutT253F was treated with AZD0530 and Imatinib for three days. Whole cell lysates were blotted for the indicated antibodies. Tubulin was probed and used as control for equal protein loading. One representative experiment out of 3 is given. Click here for file

## References

[B1] Puccetti E Guller S Orleth A Bruggenolte N Hoelzer D Ottmann OG Ruthardt M BCR-ABL mediates arsenic trioxide-induced apoptosis independently of its aberrant kinase activity Cancer Res 2000 60 13 3409 13 10910048

[B2] Ottmann OG Wassmann B Treatment of Philadelphia chromosome-positive acute lymphoblastic leukemia Hematology Am Soc Hematol Educ Program 2005 118 22 16304368 10.1182/asheducation-2005.1.118

[B3] Druker BJ Imatinib as a paradigm of targeted therapies Adv Cancer Res 2004 91 1 30 15327887 10.1016/S0065-230X(04)91001-9

[B4] Nimmanapalli R Bhalla K Mechanisms of resistance to imatinib mesylate in Bcr-Abl-positive leukemias Curr Opin Oncol 2002 14 6 616 20 12409651 10.1097/00001622-200211000-00005

[B5] Wilson MB Schreiner SJ Choi HJ Kamens J Smithgall TE Selective pyrrolo-pyrimidine inhibitors reveal a necessary role for Src family kinases in Bcr-Abl signal transduction and oncogenesis Oncogene 2002 21 53 8075 88 12444544 10.1038/sj.onc.1206008

[B6] Danhauser-Riedl S Warmuth M Druker BJ Emmerich B Hallek M Activation of Src kinases p53/56lyn and p59hck by p210bcr/abl in myeloid cells Cancer Res 1996 56 15 3589 96 8758931

[B7] Klejman A Schreiner SJ Nieborowska-Skorska M Slupianek A Wilson M Smithgall TE Skorski T The Src family kinase Hck couples BCR/ABL to STAT5 activation in myeloid leukemia cells Embo J 2002 21 21 5766 74 12411494 10.1093/emboj/cdf562 PMC131059

[B8] Nimmanapalli R O'Bryan E Huang M Bali P Burnette PK Loughran T Tepperberg J Jove R Bhalla K Molecular characterization and sensitivity of STI-571 (imatinib mesylate, Gleevec)-resistant, Bcr-Abl-positive, human acute leukemia cells to SRC kinase inhibitor PD180970 and 17-allylamino-17-demethoxygeldanamycin Cancer Res 2002 62 20 5761 9 12384536

[B9] Donato NJ Wu JY Stapley J Gallick G Lin H Arlinghaus R Talpaz M BCR-ABL independence and LYN kinase overexpression in chronic myelogenous leukemia cells selected for resistance to STI571 Blood 2003 101 2 690 8 12509383 10.1182/blood.V101.2.690

[B10] Martinelli G Soverini S Rosti G Baccarani M Dual tyrosine kinase inhibitors in chronic myeloid leukemia Leukemia 2005 19 11 1872 9 16179913 10.1038/sj.leu.2403950

[B11] O'Hare T Walters DK Stoffregen EP Sherbenou DW Heinrich MC Deininger MW Druker BJ Combined Abl inhibitor therapy for minimizing drug resistance in chronic myeloid leukemia: Src/Abl inhibitors are compatible with imatinib Clin Cancer Res 2005 11 19 Pt 1 6987 93 16203792 10.1158/1078-0432.CCR-05-0622

[B12] Warmuth M Simon N Mitina O Mathes R Fabbro D Manley PW Buchdunger E Forster K Moarefi I Hallek M Dual-specific Src and Abl kinase inhibitors, PP1 and CGP7 inhibit growth and survival of cells expressing imatinib mesylate-resistant Bcr-Abl kinases Blood 6030 101 2 664 72 10.1182/blood-2002-01-0288 12393636

[B13] Beissert T Hundertmark A Kaburova V Travaglini L Mian AA Nervi C Ruthardt M Targeting of the N-terminal coiled coil oligomerization interface by a helix-2 peptide inhibits unmutated and imatinib-resistant BCR/ABL Int J Cancer 2008 122 12 2744 52 18366061 10.1002/ijc.23467

[B14] Zheng X Beissert T Kukoc-Zivojnov N Puccetti E Altschmied J Strolz C Boehrer S Gul H Schneider O Ottmann OG Hoelzer D Henschler R Ruthardt M Gamma-catenin contributes to leukemogenesis induced by AML-associated translocation products by increasing the self-renewal of very primitive progenitor cells Blood 2004 103 9 3535 43 14739224 10.1182/blood-2003-09-3335

[B15] Sternsdorf T Puccetti E Jensen K Hoelzer D Will H Ottmann OG Ruthardt M PIC-1/SUMO-1-modified PML-retinoic acid receptor alpha mediates arsenic trioxide-induced apoptosis in acute promyelocytic leukemia Mol Cell Biol 1999 19 7 5170 8 10373566 10.1128/mcb.19.7.5170 PMC84360

[B16] Koyama N Koschmieder S Tyagi S Portero-Robles I Chromic J Myloch S Nurnberger H Rossmanith T Hofmann WK Hoelzer D Ottmann OG Inhibition of phosphotyrosine phosphatase 1B causes resistance in BCR-ABL-positive leukemia cells to the ABL kinase inhibitor STI571 Clin Cancer Res 2006 12 7 Pt 1 2025 31 16609011 10.1158/1078-0432.CCR-04-2392

[B17] Warmuth M Bergmann M Priess A Hauslmann K Emmerich B Hallek M The Src family kinase Hck interacts with Bcr-Abl by a kinase-independent mechanism and phosphorylates the Grb2-binding site of Bcr J Biol Chem 1997 272 52 33260 70 9407116 10.1074/jbc.272.52.33260

[B18] Nowak D Boehrer S Hochmuth S Trepohl B Hofmann W Hoelzer D Hofmann WK Mitrou PS Ruthardt M Chow KU Src kinase inhibitors induce apoptosis and mediate cell cycle arrest in lymphoma cells Anticancer Drugs 2007 18 9 981 95 17704648 10.1097/CAD.0b013e3281721ff6

