# Supplementary figures and images for: The effect of the dual Src/Abl kinase inhibitor AZD0530 on Philadelphia positive leukaemia cell lines
Source: BMC Cancer. 2009 Feb 13;9:53. doi: 10.1186/1471-2407-9-53 (PMC2654659; doi:10.1186/1471-2407-9-53)

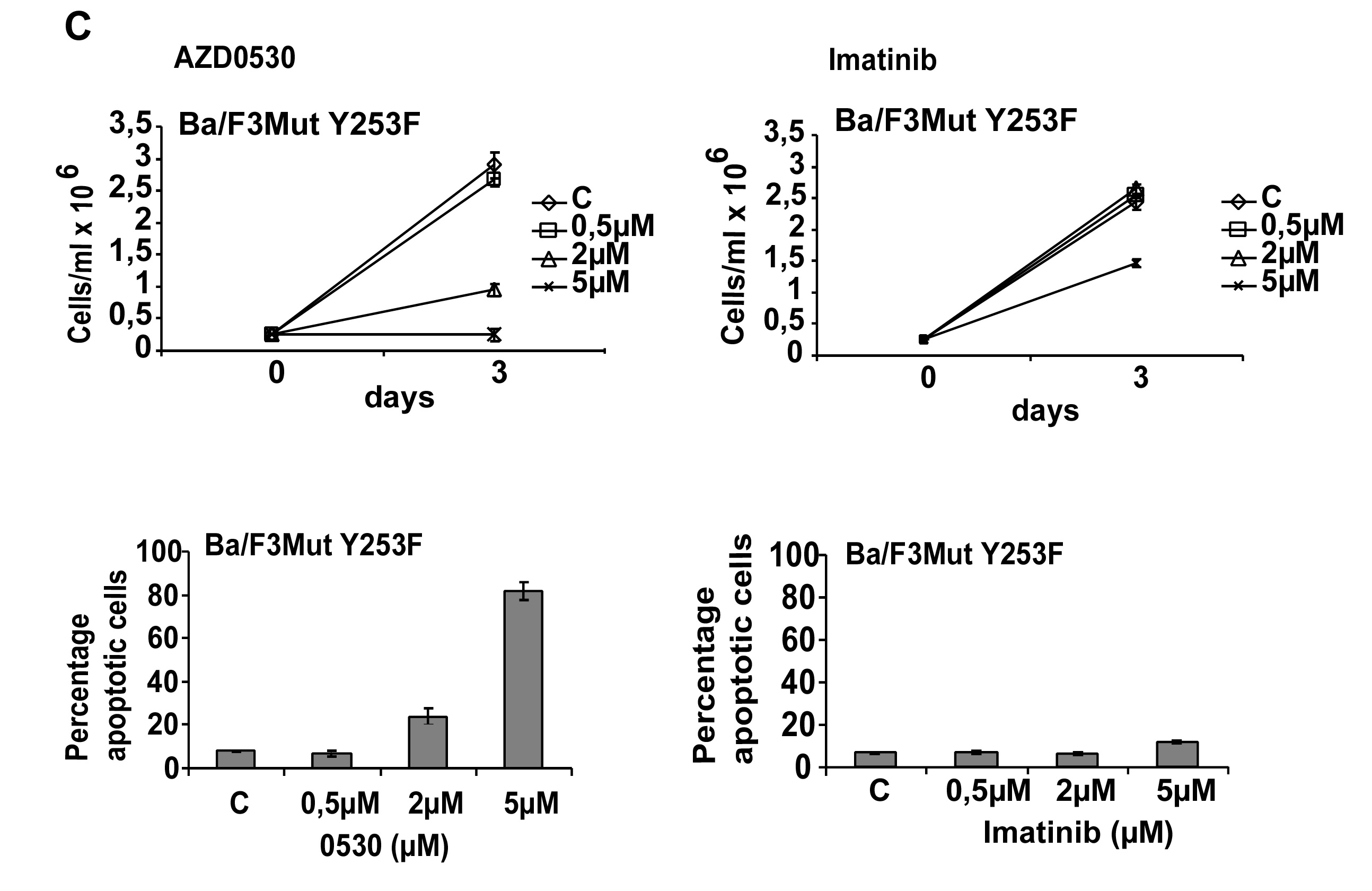

Supplement: Additional file 1 — AZD0530 overcomes Imatinib resistance and induces growth arrest in Imatinib resistance Ba/F3MutT253F cells. Ba/F3MutT253F cells were grown in the presence of AZD0530 (left) and Imatinib (right) for three days. Proliferation was assessed by trypan blue exclusion of viable cells, and the percentage of apoptotic cells was measured by staining with 7-AAD. Results are the mean of 3 independent experiments carried out in duplicates +/- S.D. [file 1471-2407-9-53-S1.jpeg]

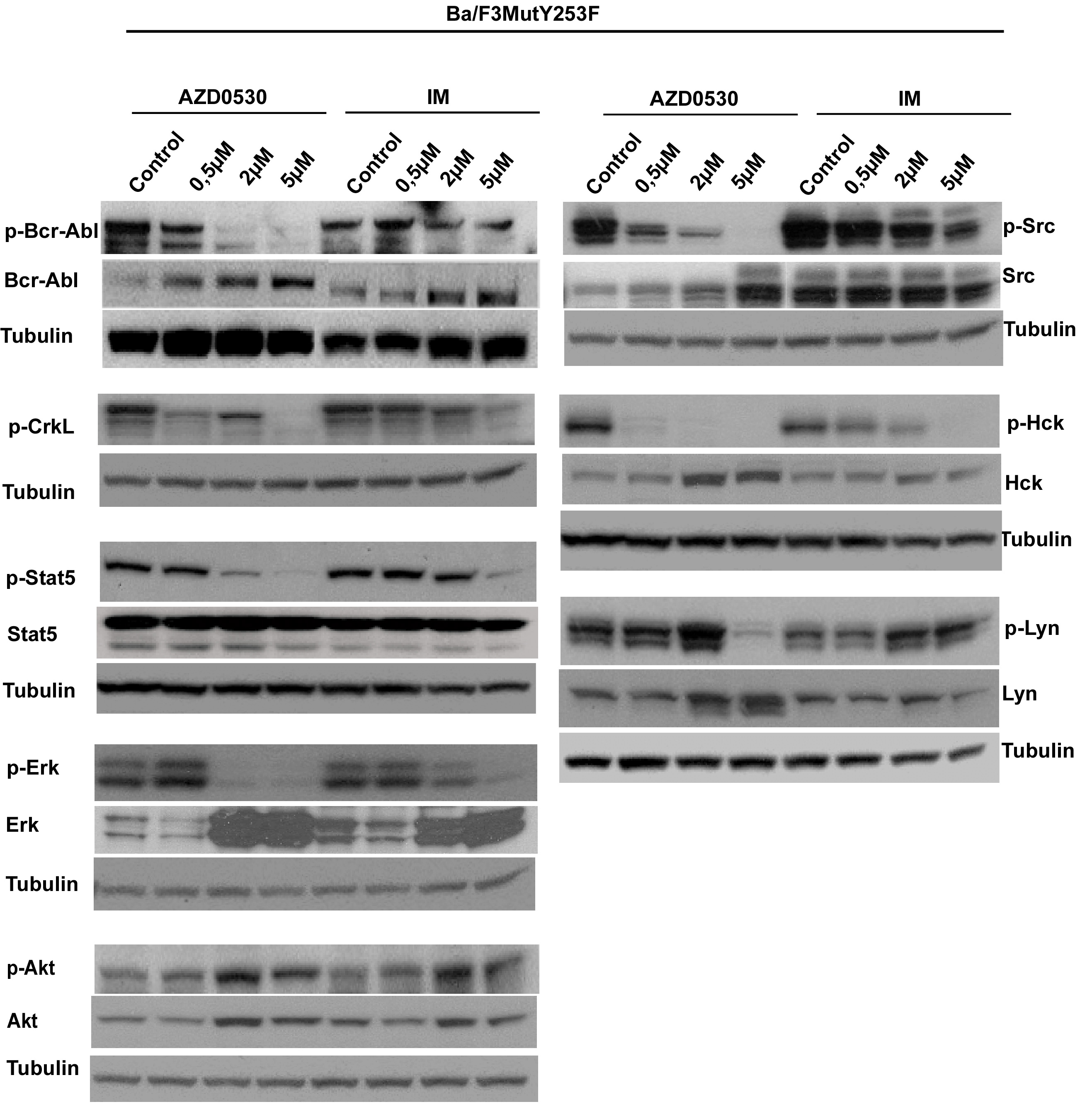

Supplement: Additional file 2 — AZD0530 inhibits Bcr-Abl activation and its downstream signalling pathways in Imatinib resistance Ba/F3MutT253F cells. The Imatinib resistant cell line Ba/F3MutT253F was treated with AZD0530 and Imatinib for three days. Whole cell lysates were blotted for the indicated antibodies. Tubulin was probed and used as control for equal protein loading. One representative experiment out of 3 is given. [file 1471-2407-9-53-S2.jpeg]
